# The Impact of Manual Ability Level on Participation of Children with Cerebral Palsy in Life Areas: A Cross-Sectional Study

**Published:** 2019

**Authors:** Marzieh PASHMDARFARD, Reza SHERVIN BADV

**Affiliations:** 1 Department of Occupational Therapy, School of Paramedical and Health, Zanjan University of Medical Sciences, Zanjan,Iran.; 2 Children’s Medical Center, Pediatric Center of Excellence, Tehran University of Medical Sciences, Tehran, Iran.

**Keywords:** Cerebral palsy, Manual ability, Patient participation, Occupations, Motor skills

## Abstract

**Objectives:**

Participation is a complex and context-dependent concept, which several factors can influence it. The aim of this study was assessing the relationship between the upper extremity function level of children with cerebral palsy (all type of cerebral palsy and severity) and their participation in different life areas.

**Materials & Methods:**

This cross-sectional study assessed the relationship between the level of upper extremity function of cerebral palsy children and their participation in different life areas. Participants were 274 parents of children with cerebral palsy of the schools of children with special needs and occupational therapy clinics in Tehran, Iran in 2018. They completed the Manual Ability Classification System (MACS) to determine the level of upper extremity function of children with cerebral palsy and Children Participation Assessment Scale-Parent version (CPAS-P) (to determine the participation level of children with cerebral palsy) questionnaires separately.

**Results:**

The mean age of children was 8 yr and 8 months old (at least 6 yr and maximum 12 yr). The correlation between the level of upper extremity function and the overall score of each dimension of participation is significant (*P*<0.05) and moderate.

**Conclusion:**

The upper extremity function of children with cerebral palsy has a moderate and significant relationship with the participation of children with cerebral palsy in different life areas and with different dimensions of participation especially parental satisfaction dimension. Therefore, there is a correlation between upper extremity function and participation in occupations, but this relationship is moderate and is not very strong.

## Introduction

Cerebral palsy (CP) is a non-progressive neurological disorder in the brain that results in abnormal muscle tone, poor balance, poor muscle control, and limitation in the performance and participation of the individual in activities (1). It is the most important factor in the physical disability of children in developed countries (2). The prevalence of this disorder is 2 and 2.6 per 1,000 live births in the world (3) and in Iran, repectively (4). 

The focus of health system interventions on these individuals is to reduce existing deficiencies and to increase the ability to perform daily activities independently as possible as they can (5). However, according to the International Classification of Functioning (ICF) over the past decade, the health system's attention should be focused on aspects that cover a broader understanding of health that this holistic and all-embracing attitude is the same as the participation of individuals with any impairment in all life areas (6). According to ICF, participation is involvement in life situation (7). 

Participation in purposeful activities has physically and mentally acceptable benefits to individuals (8). The pattern of participation of children with CP in occupations is different from that of their normal peers and has less diversity and less intensity in participation (4, 9-13). Since participation is a complex and context-dependent concept, several factors can influence it. Among these, personal factors associated with an individual have a more significant impact on the level of individuals’ participation (14). Personal factors influencing the participation of children in leisure activities. Factors such as age, gender, upper and lower extremity function, family socioeconomic status, parenting education and the like can be one of the factors that influence the extent of children participating in leisure activities (14, 15).

However, there was no study assessed the impact of personal factors on participation in all areas of occupation. Since in occupational therapy, further interventions emphasize the improvement of the functional level of the upper extremities, and especially in those with physical problems, therapists focus their interventions on the upper extremities most of all and there is always the assumption among occupational therapists that to perform various activities the ability of the upper extremity is essential. 

However, there was no study that examined this hypothesis in all life areas. Therefore, this study was designed for assessing the relationship between the level of upper extremity function of children with CP and their participation in different life areas.

## Materials & Methods

This cross-sectional study has assessed the relationship between the level of upper extremity function of CP children and their participation in different life areas. Sampling was done in the schools of children with special needs and occupational therapy clinics in Tehran, Iran, in 2018. 

The project was initially approved at the Zanjan University of Medical Sciences with the code of ethics ZUMS.REC.1396.07 then a letter was sent from this university to go to the schools of children with special needs. The children that had the inclusion criteria were enrolled into the study and then the parents of children with CP completed the consent form. 

The sampling method was the convenience sampling method. The inclusion criteria were the child's age range is 6-12 yr old, a child is diagnosed with CP (all type of CP, IQ>70) by a pediatric neurologist or neurologist; parents of children with CP are literate to read and write to complete the questionnaires.

The exclusion criteria were: Presence of epilepsy or resistant seizures in the child, having other diseases such as neuromuscular disorder in children, having BTX injections and orthopedic surgery in the last 12 months, and the blindness and deafness in children.

***Participants***

Overall, 274 parents of 6-12 yr old children with CP completed both the Manual Ability Classification System (MACS) and the Children Participation Assessment Scale-Parent version (CPAS-P) by self-report. 

**Outcome Measurement**

For data collection, MACS and CPAS-P questionnaires were used. 

**MACS**

To determine the level of upper extremity function of children with CP the MACS questionnaire was used (16). Validity and reliability of this questionnaire have been assessed by the test-retest reliability of this questionnaire for the total score has been obtained ICC=0.92 (17). The MACS questionnaire is a standard observational classification system that categorizes children with CP on the basis of current manual ability, limitation of manual abilities and the need for technology and auxiliary devices in five levels. Therefore, Level 1 shows maximum independence in manual abilities and Level 5 shows minimum independence in manual abilities (Level **I**: Manually manipulates objects easily and successfully with some limitations in speed and accuracy. Level **V**: Does not manipulate objects and need full help to perform simple functions). This test is a valid test and is easy to use and widely used globally (16).

***CPAS-P***

In order to collect information about the participation of children with CP, the CPAS-P questionnaire developed in Iran was used (18, 19). The CAS-P questionnaire has 71 items that include 71 items in 8 sub-categories consisting of Activity of Daily Living (ADL: 11), Instrumental Activity of Daily Living (IADL: 12), play (13), leisure (16), education (4), work (2), social participation (12), rest/ sleep (3). This questionnaire is a parent report scale that assesses each item from five dimensions of diversity, intensity, with whom, enjoyment, and parental satisfaction.

***Statistical analysis***

For data analyzing the SPSS (Ver.21, Chicago, IL, USA) was used. To determine the normal distribution of data, the Kolmogorov-Smirnov test (K-S) and subsequently to measure the relationship between variables Spearmen correlation test was used. The Spearman correlation score value is as follows: 0-20 (very low correlation), 20-40 (low correlation), 40-60 (moderate correlation), and 60-80 (high correlation), and 80-100 (very high correlation) (20).

## Results

The mean age of children participating was 8.8 yr old (at least 6 and maximum 12) (Table 1).

**Table 1:** Demographic characteristics of participants

**Variable**		**Frequency**	**Percent (100%)**
**Age (yr)**	6	18	6.6
	7	26	9.5
	8	43	15.7
	9	36	13.1
	10	45	16.4
	11	39	14.2
	12	67	24.5
**MACS** * ** Level**	I	57	20.8
	II	79	28.8
	III	49	17.9
	IV	42	15.3
	V	47	17.2
**Gender**	Male	171	62.4
	Female	103	37.6
**Type of **CP	Hemiplegia	41	15.0
	Diplegia	95	34.7
	Quadriplegia	78	28.5
	Atetoid	14	5.1
	Ataxi	25	9.1
	Dystonic	21	7.7

* MACS; Manual Ability Classification System

The results of the correlation between the upper extremity function level of children with CP and their participation in the different domains of occupation are presented in Table 2. The correlation between the level of upper extremity function and the overall score of each dimensions of participation was significant (*P*<0.05) and moderate correlation (Table 2).

**Table 2:** The Spearman correlation between MACS and participation in life area

**Area of occupation**	**Objective dimension**	**Means**	**SD**	**R**	***P***
Total score	Diversity	34.43	15.11	-0.46	**0.00**
	Intensity	139.90	63.78	-0.43	**0.00**
	With whom	68.59	32.46	-0.41	**0.00**
	Enjoyment	124.70	61.81	-0.41	**0.00**
	Parent satisfaction	104.47	52.10	-0.49	**0.00**
Activity of Daily Living(ADL)	Diversity	8.22	3.14	-0.33	**0.00**
	Intensity	43.38	17.93	-0.31	**0.00**
	With whom	14.74	6.04	-0.01	**0.77**
	Enjoyment	27.02	12.08	-0.34	**0.00**
	Parent satisfaction	24.16	10.75	-0.44	**0.00**
Instrumental Activity of Daily Living(IADL)	Diversity	5.00	3.10	-0.54	**0.00**
	Intensity	22.87	15.26	-0.52	**0.00**
	With whom	9.45	6.31	-0.50	**0.00**
	Enjoyment	18.18	11.98	-0.54	**0.00**
	Parent satisfaction	15.39	10.42	-0.57	**0.00**
Play	Diversity	5.30	3.58	-0.37	**0.00**
	Intensity	18.58	14.82	-0.34	**0.00**
	With whom	11.97	9.13	-0.35	**0.00**
	Enjoyment	19.82	14.13	-0.38	**0.00**
	Parent satisfaction	16.07	11.23	-0.40	**0.00**
Leisure	Diversity	7.88	3.97	-0.31	**0.00**
	Intensity	25.51	15.11	-0.25	**0.00**
	With whom	15.95	9.05	-0.27	**0.00**
	Enjoyment	30.21	16.73	-0.25	**0.00**
	Parent satisfaction	24.24	13.81	-0.34	**0.00**
Social Participation	Diversity	11.65	8.87	-0.27	**0.00**
	Intensity	10.32	7.07	-0.31	**0.00**
	With whom	17.47	12.55	-0.26	**0.00**
	Enjoyment	14.26	10.27	-0.31	**0.00**
	Parent satisfaction	0.51	0.91	-0.027	**0.63**
Education	Diversity	1.65	3.04	-0.028	**0.62**
	Intensity	1.17	2.36	-0.023	**0.69**
	With whom	1.83	3.29	-0.017	**0.77**
	Enjoyment	1.56	2.92	-0.030	**0.60**
	Parent satisfaction	0.74	0.55	-0.19	**0.001**
Work	Diversity	4.00	3.03	-0.19	**0.001**
	Intensity	1.41	1.20	-0.12	**0.03**
	With whom	2.46	2.05	-0.21	**0.00**
	Enjoyment	2.20	1.82	-0.27	**0.00**
	Parent satisfaction	2.13	1.03	-0.22	**0.00**
Sleep/ rest	Diversity	12.20	6.29	-0.22	**0.00**
	Intensity	3.54	2.06	-0.11	**0.04**
	With whom	7.68	4.26	-0.27	**0.00**
	Enjoyment	6.56	3.63	-0.30	**0.00**

## Discussion

Occupational therapists are one of the most important members of the health system and rehabilitation team, which plays a key role in improving the functioning of children with CP. In dealing with patients with physical problems, the main focus of occupational therapists is to improve the upper extremity function of children with CP to promote their participation in different life areas. 

The results of this study showed that the upper extremity function of children with CP was moderately correlated with the total score of their participation in different life areas. In this study, the highest correlation was found between the upper extremity function of children with CP and their participation in Instrumental Activities of Daily Living (IADL) (0.5 <r <0.6) and the least correlation was found between upper extremity function of children with CP and their participation in activities related to work and Sleep/Rest (r <0.2). One of the most important achievements of this study was about the moderate correlation between the dimensions of participation and the upper extremity function of children with CP in mobility based activities. The highest relationship was related to upper extremity function of children with CP with parental satisfaction, which by increasing the function of upper extremity of children with CP for participation in all aspects of life areas the parental satisfaction dimension increased more than other dimensions. Therefore, the total score of upper extremity function of children with CP and parental satisfaction dimension is more relevant than other dimensions of participation (diversity, intensity, with whom, enjoyment). The parent report qualities of life of children with CP are related to the Gross Motor Function Level (GMFL) of their children with CP (21). The most important things in reporting the ability of children with CP in their parent opinion is the level of the children abilities especially the ability of gross and fine motor functions to participating in ADL and IADL activities (22). When the ability of children in gross motor function and fine motor function improve the independence of the children for participation in different life areas will improve too and the caregiving burden reduces for parents (23).

 Children with CP that have the history of seizure, inappropriate language, and poor educational level, are more likely to be faced with problems related to social participation and These individual factors can limit the involvement of these children in social participation (24). The severity of a disability is one of the most important factors influencing the participation of children with CP in leisure activities (25). 

In the present study, if the upper extremity function of children with CP be better and improves (the severity of the disability is reduced); the participation of the children with CP in different life areas will improve too. The personal preferences of individuals in choosing leisure activities were among the factors that determine the intensity and diversity of a person's participation in leisure activities (5). In the present study, the upper extremity function of children with CP has a significant relationship with all dimensions of participation, especially parental satisfaction dimension. Children with CP due to their upper extremity function impairment had problem in performing the activities related to ADL, and improving the upper extremity function of these children could improve their participation in the activities related to ADL (26). This study was the first study indicated the relationship between motor components (Manual Ability) of children with CP and their participation in occupational performances. Most studies showed the impact of motor components on performance skills and functions of children with CP (25, 26). The results of present study showed that the motor components of children with CP have significant correlation with their participation in occupational performances especially the performance which needs mobility and motor skills. 

The impact of variables such as level of intelligence, the level of gross motor function, the use of assistive device, the type of CP and the ability of the lower extremity function on participation in different life areas of children with CP were not assessed.

**In conclusion, **the upper extremity function of children with CP has a moderate and significant relationship with the participation of children with CP in different life areas, and with different dimensions of participation especially parental satisfaction dimension. Therefore, improving the upper extremity function of children with CP promotes their participation moderately so, promoting manual ability in addition to the other factors such as caregivers training, Fitting the environment, etc. could be improved to promote the participation of children with CP.
